# Antibacterial activity, bio-compatibility and osteogenic differentiation of graphene oxide coating on 3D-network poly-ether-ether-ketone for orthopaedic implants

**DOI:** 10.1007/s10856-021-06614-7

**Published:** 2021-10-26

**Authors:** Cui Guo, Ran Lu, Xin Wang, Su Chen

**Affiliations:** Laboratory of Biomaterials and Biomechanics, Beijing Key Laboratory of Tooth Regeneration and Function Reconstruction, School of Stomatology, Capital Medical University, Tiantan Xili No.4, Beijing, 100050 People’s Republic of China

## Abstract

Poly-ether-ether-ketone (PEEK) has attracted increasing attention as a promising orthopaedic implant material owing to its excellent mechanical properties and biocompatibility. However, its antibacterial properties must be improved as an implant material. In this study, PEEK was sulfonated to obtain a porous surface, and graphene oxide (GO) was deposited to form a coating with antibacterial activity and biocompatibility. After PEEK was sulfonated for different durations, GO was deposited on the surface to prepare the coating (SPEEK-GO), which was then characterised using scanning electron microscopy (SEM), Raman spectroscopy, and contact angle measurements. The in vitro study included antimicrobial and cellular tests. The results showed that the PEEK sulfonated using a 10-min treatment exhibited a uniform porous structure and provided a better basal surface for the deposition of GO. The SPEEK-GO coating displayed strong antibacterial activity against two common dental pathogens. It exhibited good adhesion and proliferation of MC3T3-E1. Moreover, it showed osteogenic differentiation as bone implant material.

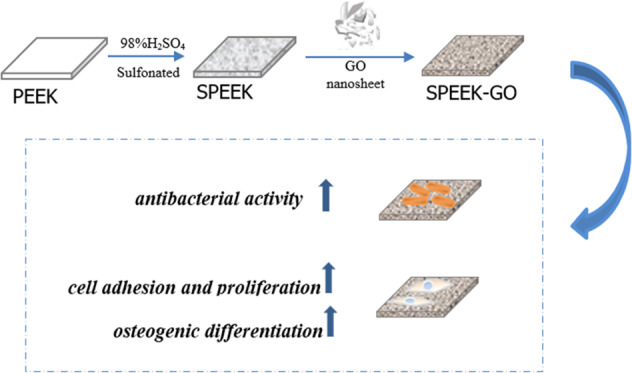

## Introduction

Titanium and titanium alloys have been traditionally used as the materials for dental implants and are still widely used in clinical applications. However, more and more research has indicated that clinical use of metallic materials poses several problems for human health. First, titanium has a high elastic modulus compared to human bone tissue, so it causes stress fatigue in the surrounding bone tissue and leads to bone absorption or loss around the implant [1]. Another disadvantage of titanium is its potential. Although the allergenicity rate of titanium is low (the reported likelihood of titanium allergies is ~0.6%), the wide clinical use of titanium-based implants would still lead to several unfavourable outcomes that limit its applications [2]. Moreover, the metallic appearance of the implant is not aesthetically pleasing. Due to these issues, an increasing number of patients refuse to allow the use of metal implant materials in the mouth or even in the body. Therefore, the development of non-metallic implants and prostheses has attracted intense research.

Poly-ether-ether-ketone (PEEK) is a non-metallic polymer that has gradually become widely used in the biomedical field because of its excellent physical and chemical properties [3]. As early as the late 1990s, PEEK replaced traditional metals as the preferred high-performance thermoplastic material for plastic and trauma surgery. In recent years, PEEK, which possesses an elastic modulus similar to that of native bone tissue [4] and can be easily formed into custom 3D printed shapes [5], has attracted increasing research attention for the development of dental applications. Using three-dimensional finite element analysis, Sarot et al. [6] found through randomised controlled experiments that there are no significant differences between the stress distributions around PEEK and titanium implants. Similarly, Koch et al. [7] found no significant difference between PEEK and titanium implants in bone resorption and soft tissue inflammation. Moreover, they found that the tendency of attachment of oral microorganisms to PEEK implants was essentially the same as that to titanium and zirconium oxides. These advantageous properties of PEEK make it a promising candidate material for the replacement of metallic materials in implants.

The inflammatory response of the tissue around an implant is believed to be the main cause of implant failure [8]. The presence of a variety of symbiotic and pathogenic bacteria in the oral cavity makes the environment around the dental implants highly susceptible to infection. Bacterial adhesion and aggregation on the surface of the implant can easily cause infection of the surrounding tissues, creating risks for long-term implant use [9]. Experimental studies have demonstrated that infection occurs with a certain probability in the surrounding tissues after dental implantation, either during the early healing of the wound or for the use of the implant in the later stage of treatment [10]. Currently, research on new titanium implants has mainly focused on the use of surface modification techniques to develop coatings that can not only improve the surface biological activity and promote the integration of implants with soft and hard tissues but also reduce the infection rate of the surrounding tissues [10, 11]. Therefore, study of the surface modification of implants through the loading of antibacterial agents has become a hot topic.

Severe overuse of traditional antimicrobial agents has led to the emergence of drug resistance, giving rise to an increasingly urgent need to identify antibacterial agents with strong antibacterial effects, not drug resistance, and few side effects. To date, antibacterial materials such as cationic metal nanoparticles and quaternary ammonium salt compounds have demonstrated strong antibacterial effects. Nevertheless, while these materials can effectively inhibit the adhesion and proliferation of bacteria, they also show disadvantages such as high cost, relatively complicated processing, and high cytotoxicity. Graphene oxide (GO) has gradually emerged as a leading alternative owing to its excellent antibacterial properties [12–14], high cellular compatibility [15], and potent antiviral activity [16]. It was also found that GO suspension creates a bactericidal effect against the most common dental pathogenic bacteria. Previous studies have shown that GO exhibits strong antibacterial activity towards *S. aureus* and *E. coli* when deposited on silicone rubber substrates, and it was found that GO is stabe in physiological and pathological conditions [17]. However, few studies have investigated GO as an antibacterial coating on PEEK for oral applications.

Cell behaviour depends on the interactions between materials and cells. Previous studies have shown that the addition of GO to a biopolymer matrix can promote cell attachment and proliferation through hydrophilic groups on the surface [18]. In addition, GO has been shown to induce differentiation of BMSCs into osteoblasts [19], and GO nanosheets have demonstrated antimicrobial activity against Gram-negative and Gram-positive bacteria [20]. These attractive properties of GO make it a promising surface modification material for guiding implant bone regeneration.

In this study, we investigated GO as a coating material on the surface of PEEK that was sulfonated to form a 3D-network-structured surface. Using this approach, we obtained a hydrophilic surface and an antibacterial coating with preliminary osteogenic potential. We believe that GO-modified biomaterials have attractive bactericidal properties to promote bone formation and would expand the application of bone tissue engineering in dentistry.

## Materials and methods

### Materials

PEEK materials with the dimensions of 10 × 10 × 1 mm^3^ were purchased from Junhua PEEK Plc (Jiangsu, China). The GO dispersion was obtained from XFNANO Tech (Nanjing, China), and 98% sulfuric acid, acetone, ethyl alcohol, and other chemicals used in this work were supplied by KeLong Chemical (Chengdu, China).

### Specimen preparation

For the preparation of sulfonated PEEK (SPEEK), PEEK was successively cleaned with acetone, ethanol, and deionised water. The samples were immersed in 98% sulfuric acid for either 5 or 10 min and then in 1 M NaOH for 5 min. Thereafter, the samples were removed and rinsed in acetone, ethanol, and deionised water, followed by ultrasonic oscillation cleaning for 5 min. This step removes most of the residual material on the surface. The samples were then dried at 60 °C in an oven for 5 min and then removed and stored until further use. The PEEK samples treated with sulfuric acid for different durations were observed by scanning electron microscopy (SEM), and the optimal treatment time was selected for use in the subsequent experiments.

**Fig. 1:**
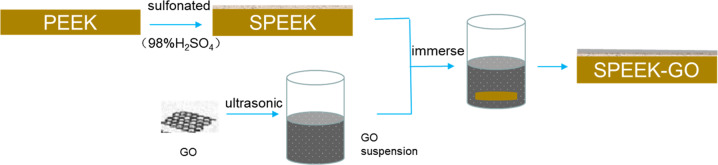
Schematic diagram showing the surface modification process of PEEK. PEEK is sulfonated with 98% H_2_SO_4_ to get SPEEK and the deposition of GO to obtain the SPEEK-GO specimens

The SPEEK specimens modified with GO (SPEEK-GO) were prepared as follows: SPEEK specimens were immersed in a GO dispersion with a concentration of 1 mg/mL for 10 min and then were removed and dried in an oven at 60 °C for 5 min. Figure 1 is the schematic diagram of PEEK surface modification process.

### Surface characterisation

The surface morphologies of the PEEK, SPEEK, and SPEEK-GO samples were observed using a Gemini SEM 500 (Gemini, Germany) field emission scanning electron microscope (FE-SEM). The parameters used for these observations were as follows:

Detector: SE2; resolution: 1.0 nm / 15 KV and 1.7 nm / 1 KV. Because the PEEK, SPEEK, and SPEEK-GO samples are conductive, the samples were coated with a thin layer of gold for 0.5 h prior to the observation. Raman spectroscopy, a widely used tool for the characterisation of carbon materials, was used in this study. The Raman spectra obtained in this study were recorded using a LabRam HR Evolution (SPM-960, Japan) laser Raman spectrometer with a laser wavelength of 633 nm. The contact angle (CA) of deionised water on the surface of the sample was measured with a video contact angle meter (OCA25, Dataphysics, Germany). Here, CA was defined as the angle between the edge of the water droplet and the surface of the material. In these measurements, deionised water droplets were deposited on the surface of the material at three different points for each specimen used for the measurements, and the average CA was calculated. These values were used to analyse the changes in the hydrophilicity and hydrophobicity of the PEEK, SPEEK, and SPEEK-GO specimen surfaces.

### Bacteria culture

A standard strain of *Porphyromonas gingivalis* (ATCC33277) was resuscitated in tryptic agar (TSA, Solarbio, China) in an anaerobic tank at 37 °C (80% N_2_, 10% CO_2_, and 10% H_2_) for 7 d. Subsequently, the recovered bacteria were cultured in an anaerobic environment at constant temperature for 7 d to obtain purified bacteria, after which a colony of the bacteria was cultured in Brian-Heart infusion (BHI, Solarbil, China) liquid medium for 48 h, and the concentration of bacterial suspension was adjusted to 1 × 10^7^ colony forming units (CFU) per millilitre for future use. The resuscitation method used for the frozen *Streptococcus mutans* standard strain (UA159) was the same as described above but involved culturing in mannitol salt agar (MSA, Solarbio, China) for 2 d, and the bacterial suspension concentration was adjusted to 1 × 10^6^ CFU/mL.

### Plate-counting experiment

The bacteria were inoculated on the surface of the samples according to the anaerobic culture requirements. Subsequently, the surfaces were rinsed gently with PBS to remove the floating bacteria. Thereafter, the specimens were transferred into a tube containing 1 mL PBS. The tube was vibrated with an oscillator to dissociate the bacteria into the solution. The bacterial suspension (100 μL) was diluted 10^3^ times and then spread evenly on BHI agar. Finally, after the completion of the culture on the agar plate, the number of bacteria was counted and analysed.

### Morphology of bacteria observed by SEM

The co-culture of the sample and bacteria was performed as described above. Thereafter, the medium was removed and discarded with a micro-sampler, and the specimens were gently rinsed with PBS three times. A 2.5% solution of glutaraldehyde (2 mL) was added to each sample and fixed overnight at 4 °C. Next, the samples were gently rinsed with PBS and dehydrated with an acetonitrile gradient. They were then dried in a vacuum, coated with a thin layer of gold, and finally observed by SEM (SU8010, Hitachi High-Technologies Corporation, Japan).

### Pathogenic genes in bacteria investigated by quantitative real-time PCR(RT-PCR)

PCR analysis was used to detect the expression of pathogenic genes in the bacteria. The bacterial culture used to obtain the samples was the same as that described above. The bacteria were collected from the samples and subjected to centrifugation. RNA was obtained through mucopolysaccharide hydrolysis, cracking thalli, deproteinisation, and centrifugation according to the Ultrapure RNA Kit (TIANGEN, China) manufacturer instructions. The obtained RNA was reverse-transcribed into cDNA using the Takara Kit (Takara, USA), and RT-PCR was implemented (Applied Biosystems 7500 Fast Real-Time PCR System, Foster City, CA, USA) with SYBR Premix Ex Taq™ (Takara, USA), using the primers and cDNA templates. As a housekeeping gene, 16S rRNA was used for calibration. The primers (5ʹ-3ʹ) used in this study are listed in Table 1. The primers were used in a volume of 20 µL per tube. The PCR thermal profile was 50 °C for 2 min and 95 °C for 10 min, followed by 40 cycles at 95 °C for 15 s, and 60 °C for 1 min. The comparative CT (2 − ΔΔCT) method was used to evaluate the gene expression differences between groups.

**Table 1:** Primer sequences for RT-PCR of oral pathogenic genes

Genes	Primer Sequences (F: forward; R: reverse)
16S rRNA	F:CCTACGGGAGGCAGCAGCAGTAG
	R:CAACAGAGCTTTACGATCCGAAA
*Fim*	F:CTGAACGAACTGCGACGCTATATGCA
	R:GTTTTTTAGTCGTTTGACGGGTCGAT
*Gtf*	F:AGCAATGCAGCCAATCTACAAAT
	R:ACGAACTTTGCCGTTATTGTCA

### Cell culture

Mouse embryonic osteoblast MC3T3-E1 cells were cultured in Dulbecco’s modified eagle’s medium (DMEML31600-500, Solarbio TECH, Beijing, China), supplemented with 1% penicillin-streptomycin solution (PB180120, Procell Life Science & Technology Co., Ltd, Wuhan, China). The cells were cultured in humidified with 5% CO_2_ at 37 °C. All samples were sterilised in an autoclave for 0.5 h before experiments. After incubation for 1–28 d, cell adhesion and proliferation were detected using a CCK-8 assay, and the osteogenesis was examined through alkaline phosphatase (ALP) activity assay and alizarin red S (ARS).

### Cell adhesion and proliferation

The MC3T3-E1 cells were seeded in 24-well plates at a density of 1 × 10^4^ cells per well. Cell adhesion and proliferation were tested using the CCK-8 kit. The cells were cultured for 1 h, 3 h, 5 h, 1 d, 3 d, 5 d, and 7 d. Samples were obtained at the corresponding time, rinsed twice with PBS, and transferred into a new plate. Next, 500 μL culture medium and 50 μL CCK-8 solution were added to each well and incubated for 2 h in the dark. The absorbance was measured at 450 nm using a microplate reader (Olympus, Japan).

### ALP assay

The ALP activity assay was performed to determine the early differentiation of MC3T3-E1 cells. The cells were seeded on sample surfaces on 48-well plates at a density of 3 × 10^4^ cells per well for 4 d in osteogenic induction medium or 5 × 10^4^ cells per well for 7 d. At each time point, the surfaces were rinsed with PBS twice and lysed with 1% Triton X-100. They were then incubated with ALP working solution, following the instructions of the ALP kit (Beyotime, Shanghai, China). The absorbance value was recorded at 540 nm with a Microplate Reader (Olympus, Japan). ALP activity was normalised against the total amount of intracellular protein standardised.

### ARS to evaluate cell mineralisation

The extracellular matrix (ECM) mineralisation of the MC3T3-E1 cells was assessed with the ARS method. The cells were seeded on different samples at a density of 5 × 10^4^ cells per well in osteogenic induction solution. After 14 d of cultivation, the cells were fixed with 4% paraformaldehyde in the dark and rinsed twice with PBS. Thereafter, the bone-like nodules were stained with ARS solution (Aladdin, Shanghai, China) for 1 h at room temperature and washed three times with PBS. Newly formed bone-like nodules on the samples are stained in dark red. Next, 10% cetylpyridine chloride solution (300 μL) was added to each well plate and shaken for 20 min. Calcium nodules on the surface of the titanium plate were dissolved, and 100 μL liquid per well was removed into the 96-well plate. The absorbance at 560 nm was measured with a microplate analyser (Fig. 1).

### Data analysis

IBM SPSS Statistics 22.0 was used for data analysis. The antibacterial performance data are presented in the form of mean ± standard deviation, and one-way analysis of variance (ANOVA) was used for inter-group comparison. Statistical significance level was accepted as *p* < 0.05.

## Results

### Material characterisation

**Fig. 2:**
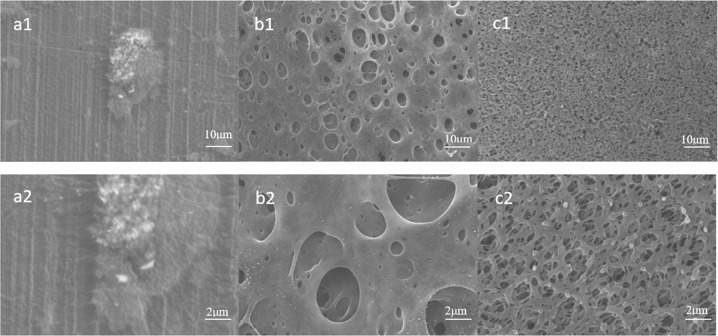
FE-SEM observations of PEEK and PEEK after sulfonation (**a**) pure PEEK; (**b**) PEEK sulfonated for 5 min; (**c**) PEEK sulfonated for 10 min

Figure 2 shows the morphologies of the pure PEEK specimen and the PEEK specimens obtained by sulfonating for 5 min and 10 min. It was observed that the surface of the PEEK specimen after polishing showed funicular scratches, while the PEEK specimen obtained after sulfonating for 5 min exhibited a pit-like appearance. The PEEK specimen obtained after sulfonating for 10 min had a spongy surface. As observed from the magnified view of the surface image (Fig. 2), the surface of this material comprised a porous 3D-network with a sponge-like structure, with a relatively uniform network of pores.

**Fig. 3:**
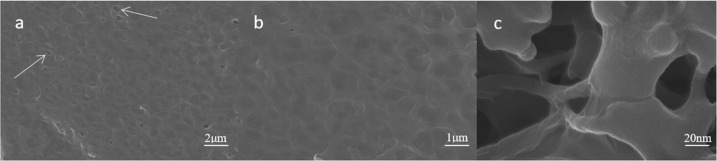
PEEK sulfonated after 10 min of specimen sedimentary GO surface morphology of (**a**) 5K; (**b**) 10K; (**c**) 50K magnification

**Fig. 4:**
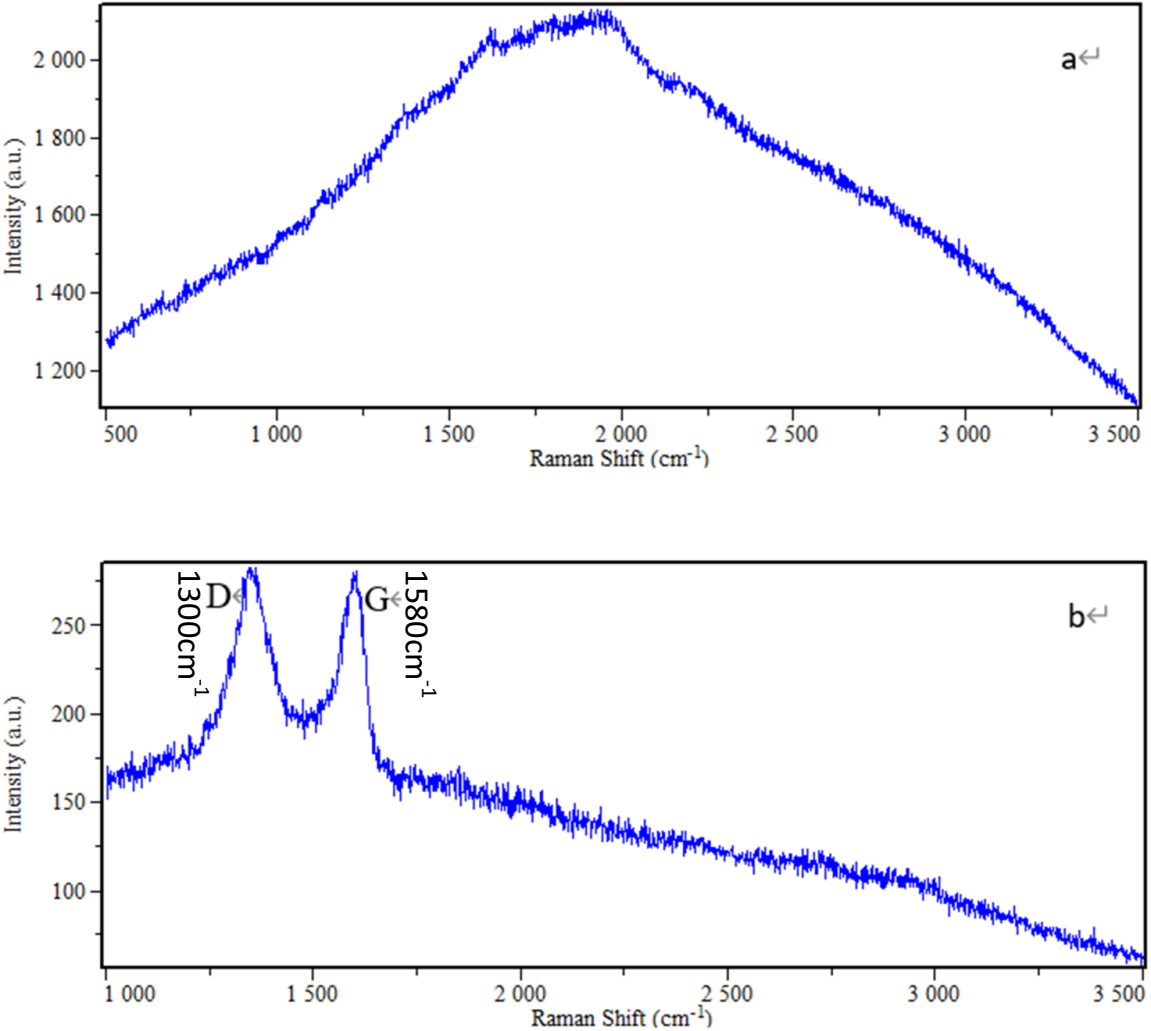
Raman spectra of PEEK and SPEEK-GO. (**a**) PEEK; (**b**) SPEEK-GO. The D-band and the G-band peaks are the characteristic Raman peaks of carbon-based crystals that appear in the vicinity of 1300 cm^−1^ and 1580 cm^−1^, respectively

Figure 3 shows the electron microscopy images at different magnifications for the samples obtained after the deposition of GO on PEEK obtained by sulfonation for 10 min. Figure 3a shows the uniform spread of GO on the SPEEK sample surface, while Fig. 3b shows that the GO nanosheets are connected into a cotton-like morphology. Figure 3c shows an enlarged view of the position indicated by the arrow in Fig. 3a. It demonstrates that the filamentous structure surface of the SPEEK material was still evenly coated by GO, and the deposited GO was also found deep inside the pores. Therefore, it was assumed that the surface of the SPEEK material was completely covered by the GO coating. Raman spectroscopy is one of the primary tools for the characterisation of carbon materials, and the D-band and the G-band peaks are the characteristic Raman peaks of carbon-based crystals that appear in the vicinity of 1300 cm^−1^ and 1580 cm^−1^, respectively. The D-band peak arises from the defects of the C atom lattice, while the G-band peak is due to the in-plane stretching vibrations of the C sp^2^ hybridised C atoms. Figure 4 shows the Raman spectra of pure PEEK and of PEEK grafted with GO after sulfonation. The D-band and G-band peaks clearly observed in the Raman spectra of SPEEK-GO showed that GO was successfully loaded onto the SPEEK surface.

**Fig. 5:**
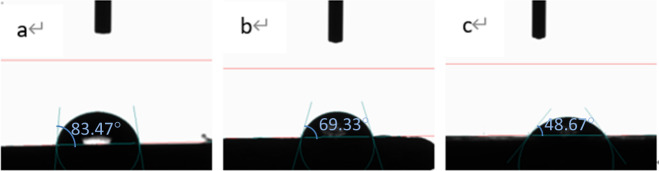
Contact angles of different material surfaces (**a**) PEEK; (**b**) SPEEK; (**c**) SPEEK-GO. The CA values of PEEK, SPEEK, and SPEEK-GO were 83.47 ± 0.15°, 69.33 ± 2.52°, 48.67 ± 1.53°, respectively

**Fig. 6:**
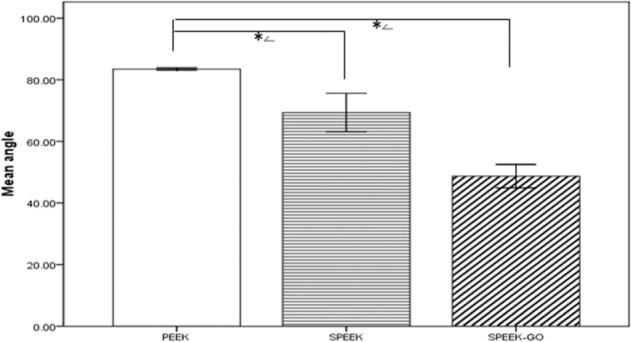
Statistical analysis of contact angles (CAs) of different material surfaces. The SPEEK-GO group was significantly different from the other two groups. (*P* < 0.01) The contact angle measurements showed that the CA values of PEEK, SPEEK, and SPEEK-GO were 83.47 ± 0.15°, 69.33 ± 2.52°, 48.67 ± 1.53°, respectively

The CAs of the surfaces of the specimens were measured using a video contact angle measuring instrument, and the hydrophilic and hydrophobic properties of the surfaces were analysed. We used the droplet method to place water droplets (4 μL) on the surface of the material and measured the CA after the droplet state was stable. Figure 5 illustrates the CA measurements of each material surface, and Fig. 6 shows a histogram of the CA values measured for the various samples.Measurements showed that the CA values of PEEK, SPEEK, and SPEEK-GO were 83.47 ± 0.15°, 69.33 ± 2.52°, 48.67 ± 1.53°, respectively. The differences between the three groups were statistically significant. It was observed that after sulfonation, the hydrophilicity of the PEEK material increased and then further improved after the loading of the GO coating on the surface.

### Antibacterial ability and adherence rate

**Fig. 7:**
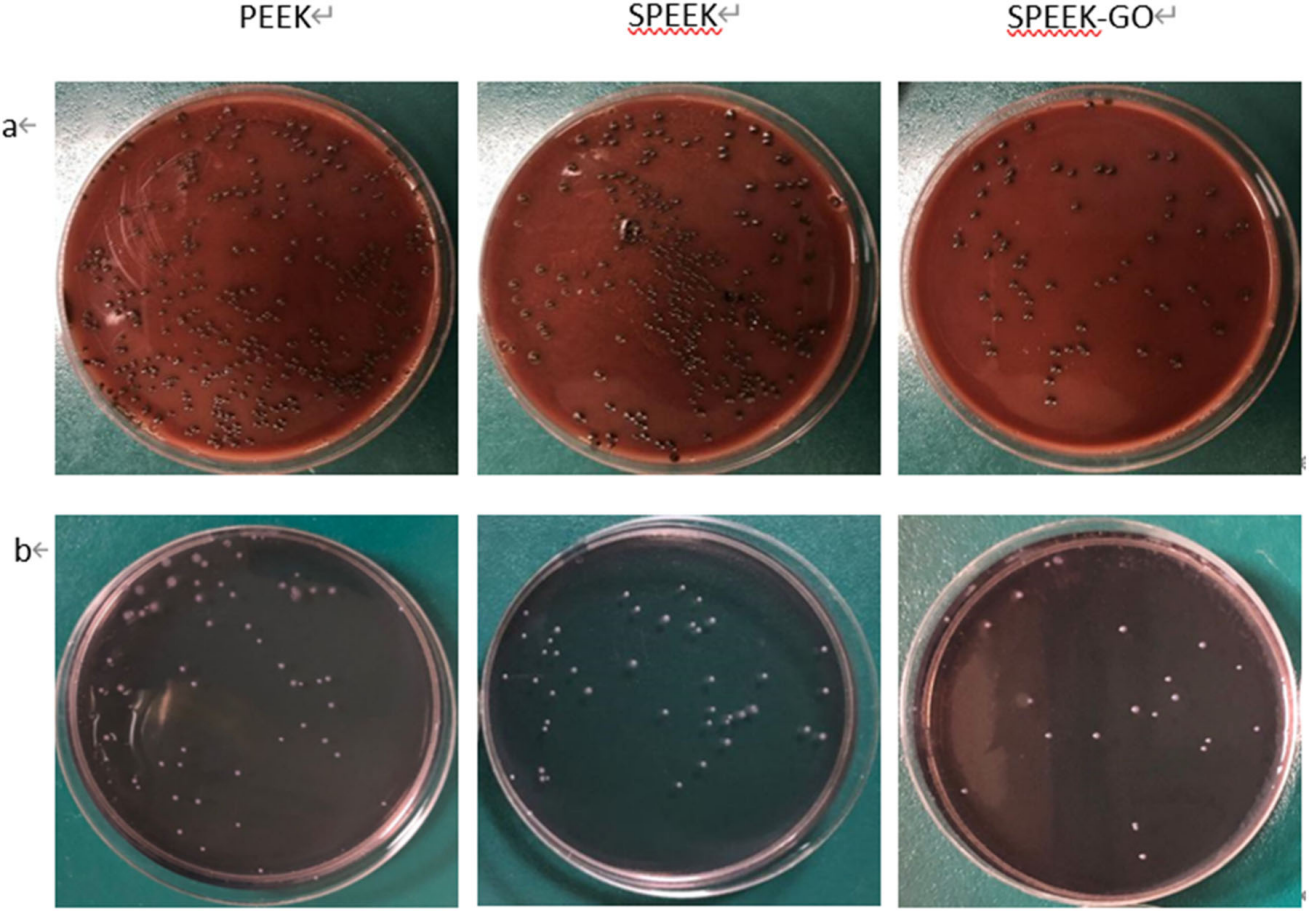
Antibacterial results of PEEK, SPEEK, and SPEEK-GO against *P. gingivalis* (**a**) and *S. mutans* (**b**). It is observed that the SPEEK-GO specimens exhibited the strongest antibacterial effect

**Fig. 8:**
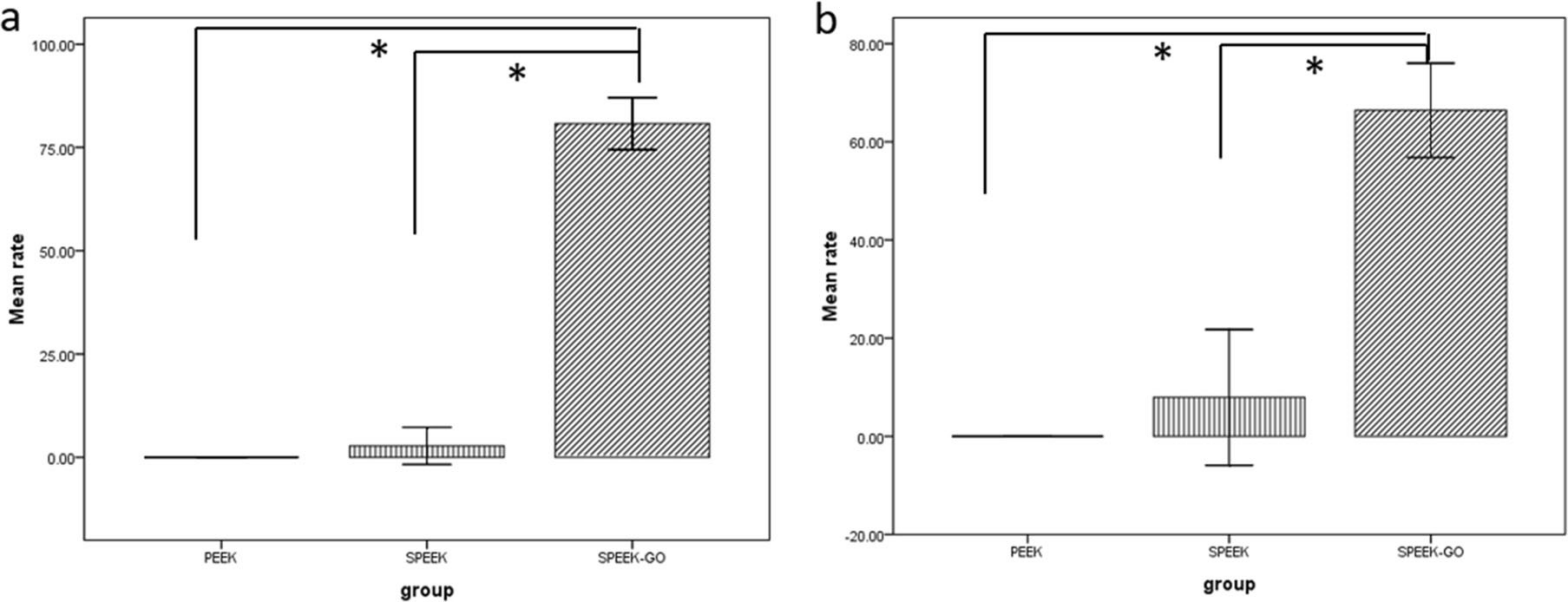
Results of rates of antibacterial activity of PEEK, SPEEK, and SPEEK-GO against *P. gingivalis* (**a**) and *S. mutans* (**b**). The SPEEK-GO bactericidal rate of *P. gingivalis* was 80.75% ± 2.54% and *S. mutans* was 66.41% ± 3.87%. The SPEEK-GO group was significantly different different from the other two groups. (*P* < 0.01)

Figure 7a shows the plate-counting results of the antibacterial activity experiment performed using *P. gingivalis* for the three groups of specimens. It is observed that the SPEEK-GO specimens exhibited the strongest antibacterial effect, with a bactericidal rate of 80.75% ± 2.54%, which was statistically significantly different from the other two groups. As shown in Fig. 7b, the antibacterial plate-counting experiment results obtained using *S. mutans* were the same as those obtained using *P. gingivalis*. The bactericidal rate of SPEEK-GO was 66.41% ± 3.87%, which was statistically significant with the other two. There was no statistical difference between the PEEK and SPEEK bactericidal rates (Fig. 8).

**Fig. 9:**
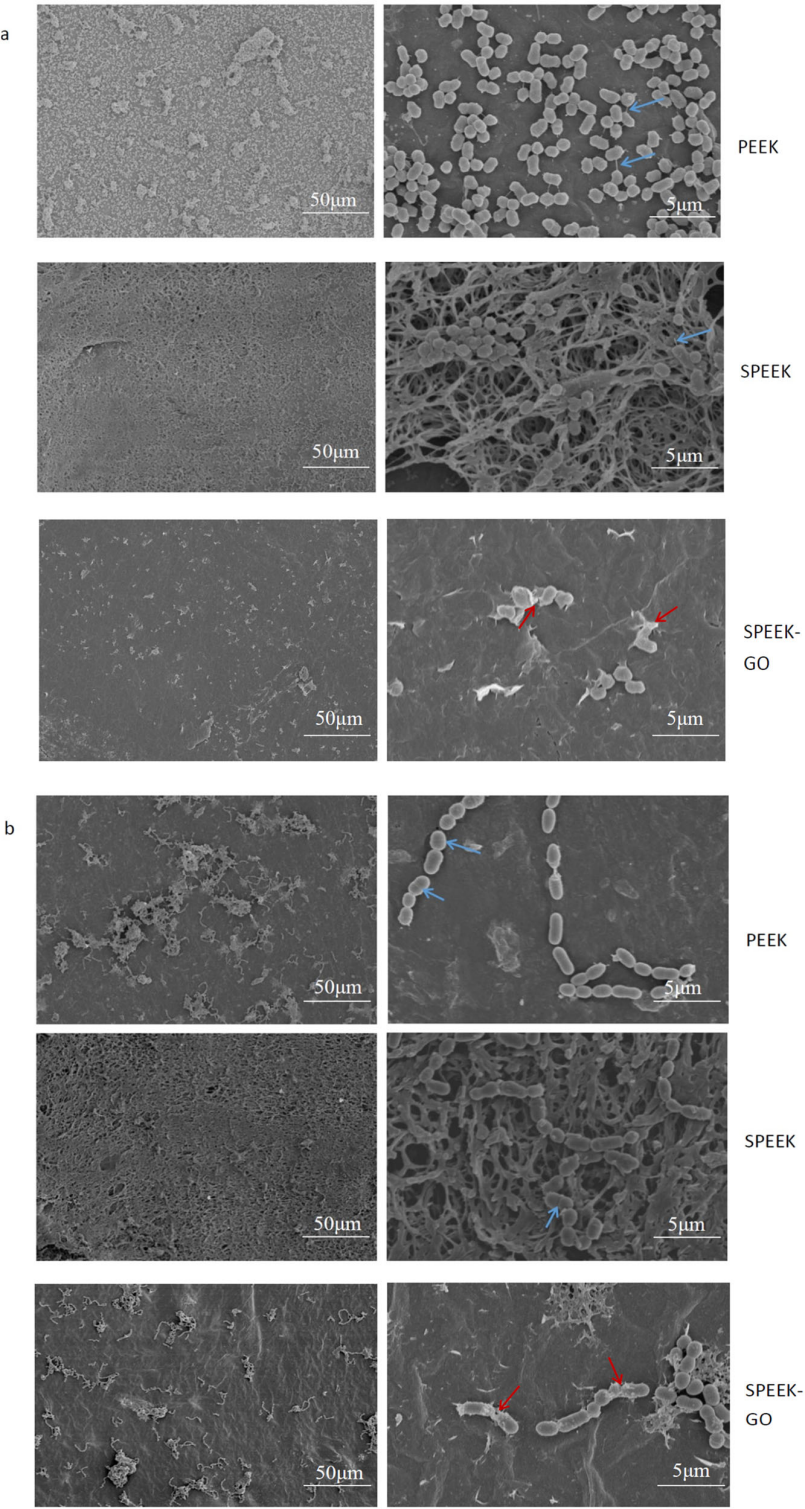
SEM image of bacterial biofilm on the surface of specimens. **a** SEM image of *P. gingivalis* on each sample; (**b**) SEM image of *S. mutans* on each sample. The blue arrows show that the bacterial cilium structure was clearly visible. The red arrows show that the bacteria appeared to shrink, and deformation and cell membrane damage were observed

The morphology of the bacteria observed by SEM provided supporting evidence for the antibacterial qualities of the SPEEK-GO coating. The GO coating had an inhibitory effect on *P. gingivalis* (Fig. 9a) and *S. mutans* (Fig. 9b). An examination of the results for *P. gingivalis* presented in Fig. 9a shows that the bacteria on the PEEK and SPEEK specimens were short rods or ellipsoids, and the bacterial cilium structure was clearly visible (as shown by the blue arrow). In contrast, a significantly lower number of bacteria were observed in the low-magnification image of the SPEEK-GO samples; the bacteria appeared to shrink, and deformation and cell membrane damage were observed at high magnification (red arrow). The antibacterial activity results for *S. mutans* are shown in Fig. 9b. The *S. mutans* on PEEK and the SPEEK samples had a round or oval shape and were connected in a chain-like manner. It was observed from the high-magnification image that the bacteria had a clear shape, a smooth and continuous surface, and no defects. A depression was observed in the centres of the cells, indicating that the bacteria were beginning to divide. In contrast, for the specimen with the SPEEK-GO coating, a decrease in the number of bacteria was observed under the low-magnification image, while in the high-magnification image, the bacteria displayed a rough appearance. It was also observed that the cell membrane was damaged, and amorphous substances were scattered around the bacteria, which may have been the cytoplasm of the bacterial cell.

**Fig. 10:**
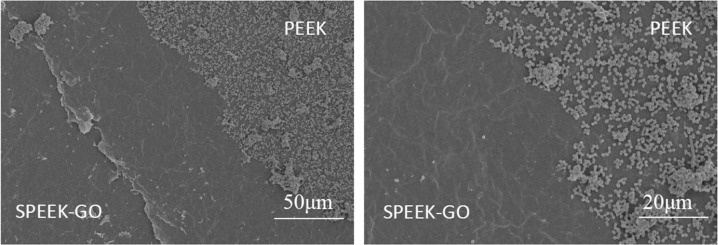
SEM image of *P. gingivalis* biofilm on the SPEEK-GO sample surface. SPEEK-GO and PEEK were placed on the same specimen for bacterial culture to reduce the operating error

Because of the operating error in the fabrication of the electron microscope samples, we used the same bacterial solution obtained from the culture for the SPEEK-GO and PEEK specimens to further verify the antibacterial effect of the surface coating. The SPEEK-GO coating is shown in the bottom left of Fig. 10. A cotton-shaped surface coating of GO on which a small amount of bacteria is scattered is clearly observed, while the image for the PEEK control group is shown at the top right that displays a number of assembled bacteria and a clear boundary line between the two different surfaces. This phenomenon was clearly observed for *P. gingivalis* but was not obvious for *S. mutans*.

### Relative gene expression

**Fig. 11:**
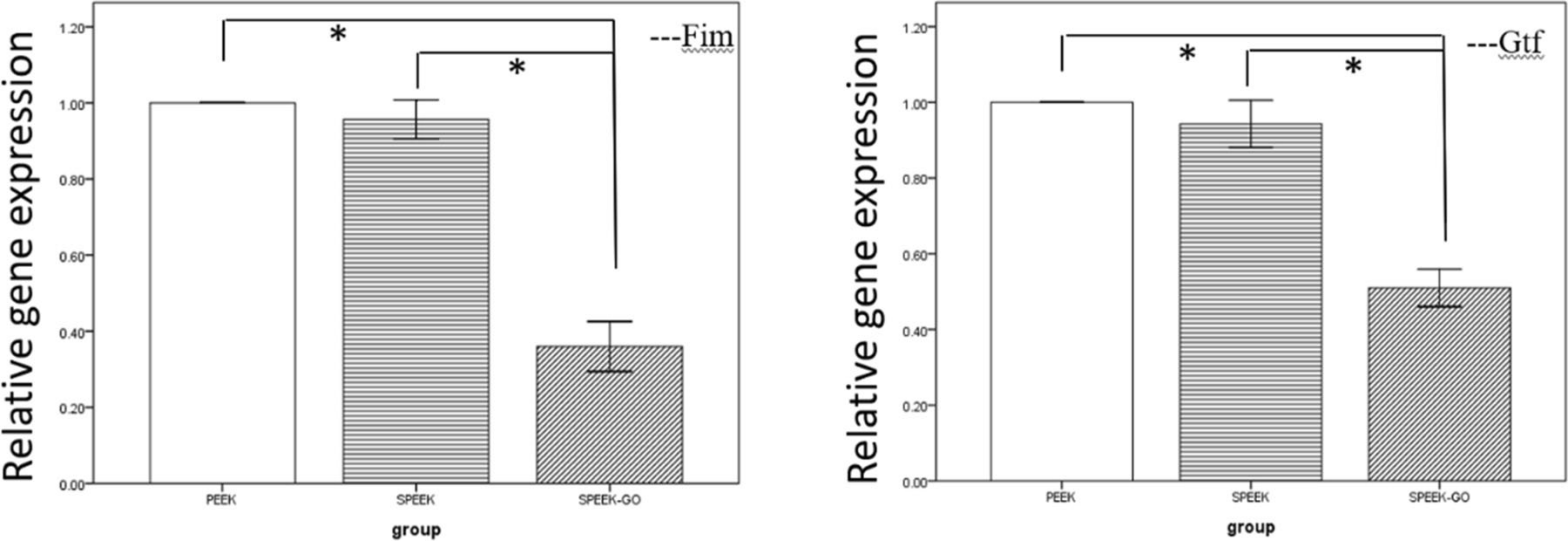
RT-PCR to evaluate the effects of gene expression. *Fim* is the pathogenic gene of *P. gingivalis* and *Gtf* is the pathogenic gene of *S. mutans*. The SPEEK-GO group was significantly different with the other two groups. (*P* < 0.01)

To determine the effect of *P. gingivalis* and *S. mutans* metabolites on the samples, the mRNA levels of the genes were assessed by quantitative RT-PCR. Figure 11 shows the results for the relative gene expression. *Fim* is the pathogenic gene of *P. gingivalis* and *Gtf* is the pathogenic gene of *S. mutans*. The results indicate that gene expression was significantly reduced in the SPEEK-GO group. The relative gene expression on SPEEK-GO showed significant statistical differences (*P* < 0.05) compared to the other two groups.

### MC3T3-E1 cell adhesion and proliferation

**Fig. 12:**
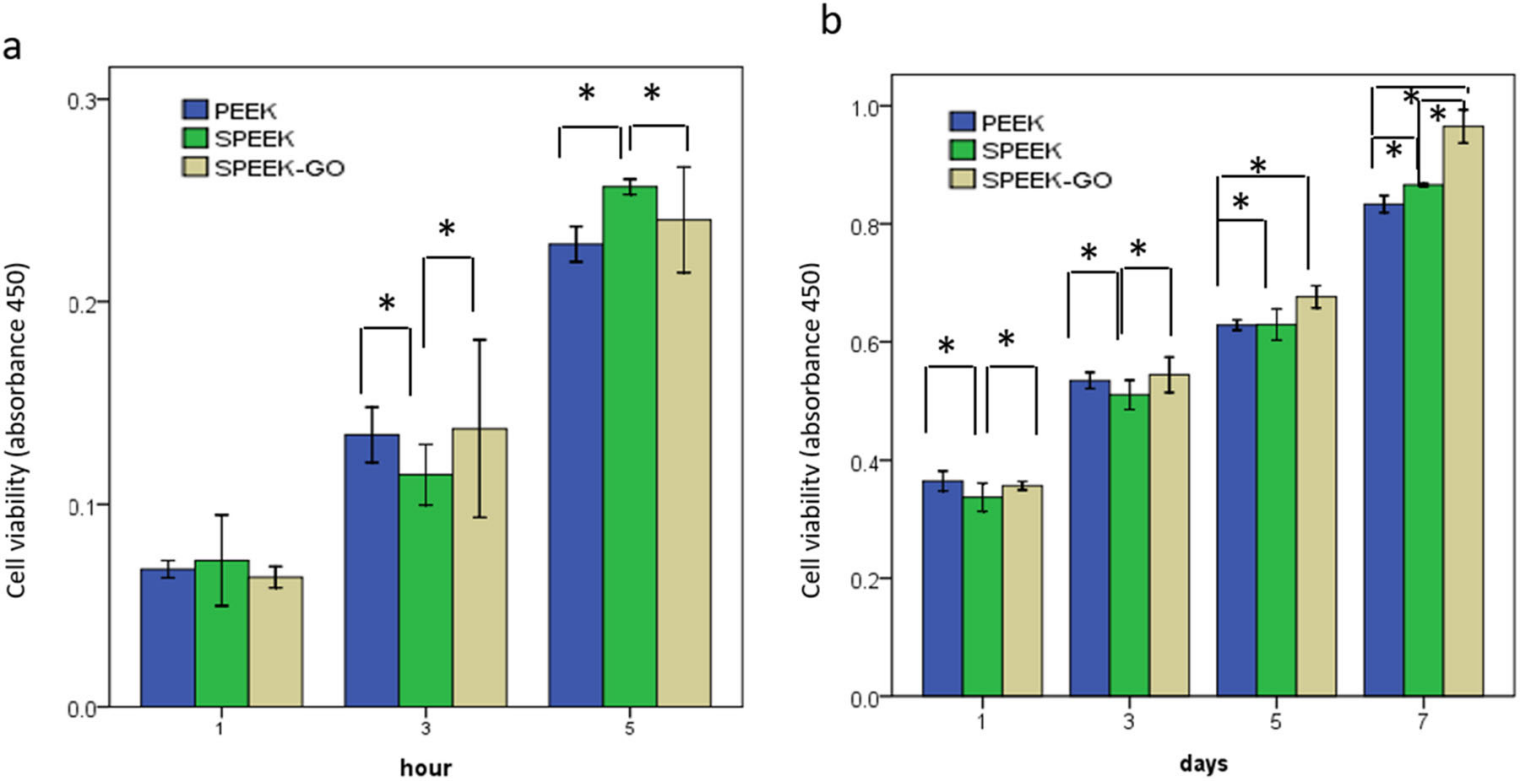
**a** Adhesion of MC3T3-E1 at 1, 3, and 5 h, as detected using CCK-8 reagent. **b** Proliferation of MC3T3-E1 cells at 1, 3, 5, and 7 d, as detected using CCK-8 reagent

Figure 12 shows the cell adhesion and proliferation capabilities assessed using the CCK-8 assay. During the adhesion stage, the cell activity of the SPEEK-GO group was kept invariable with the other two groups in the first hour. The adhesion was improved with increase in time, but the difference between the modified and control samples was not statistically significant. During the proliferation phase, there was a significant increase in the SPEEK-GO group cell activity at each time point compared to the other two groups, especially on the fifth and seventh days.

### In vitro osteogenic differentiation

**Fig. 13:**
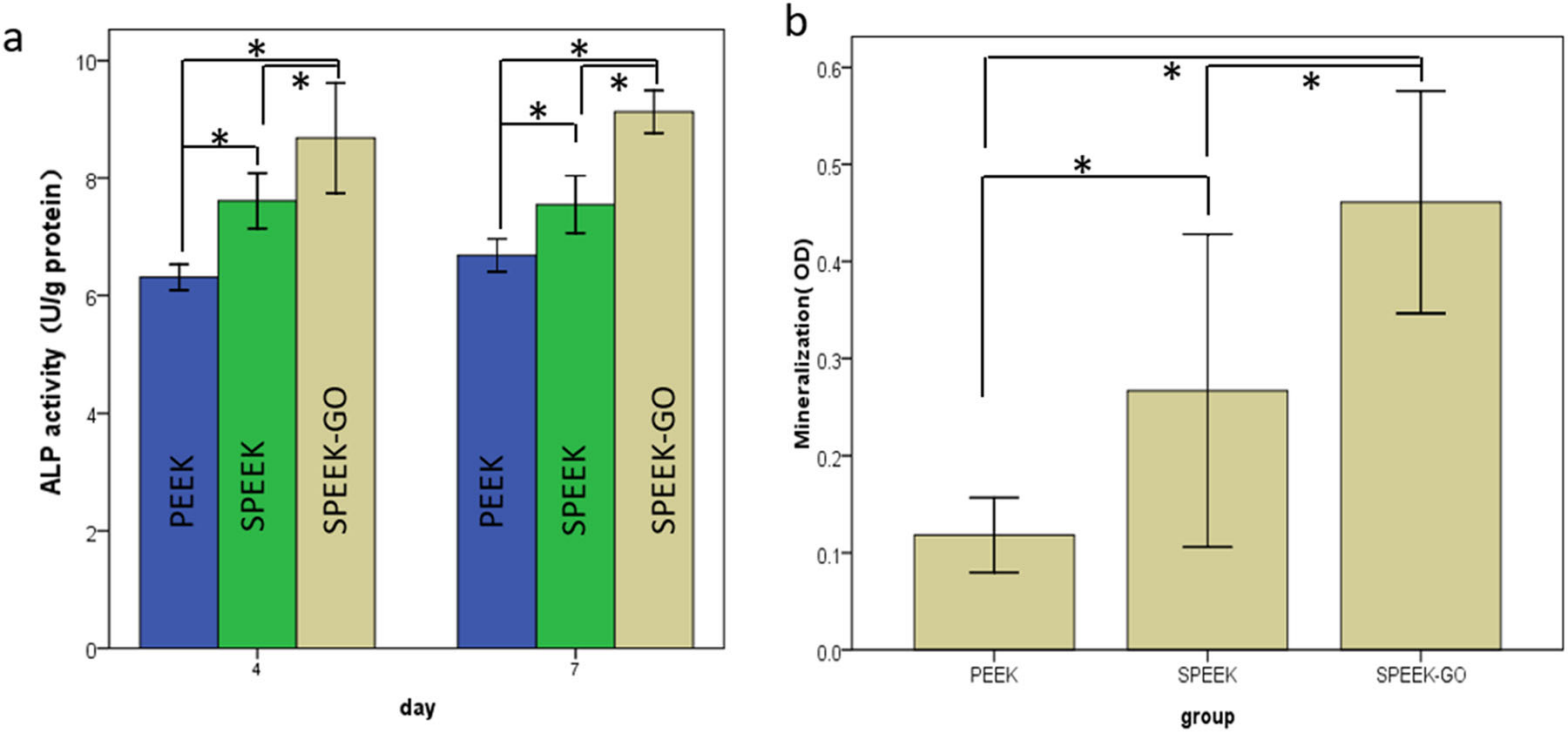
**a** Alkaline phosphatase (ALP) activity quantification at days 4 and 7 of osteogenic induction in the cells. **b** Quantification of cell ECM mineralisation on the different surfaces at 14 d

The osteogenic differentiation properties were crucial to bone regeneration, and the activity of ALP was an early marker of osteogenic differentiation. The MC3T3-E1 cells incubated with SPEEK-GO showed a higher ALP expression than those cultured with PEEK and SPEEK (*P* < 0.05). The trend became more pronounced after 7 d of incubation (Fig. 13).

**Fig. 14:**
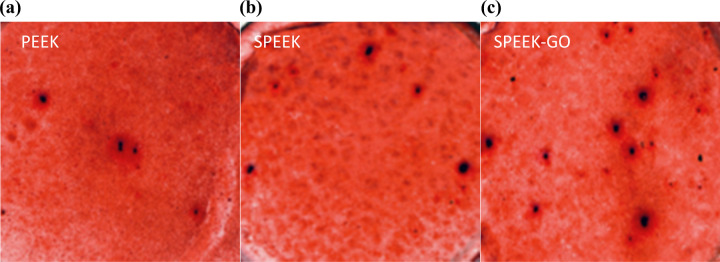
ARS of various samples (**a**) PEEK; (**b**) SPEEK; (**c**) SPEEK-GO. The SPEEK-GO group had the most calcium nodules

The authors observed ECM mineralisation, a key biological event in late osteogenic differentiation. Figure 14 shows that MC3T3-E1 cells incubated with PEEK samples displayed the weakest ECM mineralisation at day 14. The ECM mineralisation capacity of MC3T3-E1 cells grown on SPEEK-GO substrates showed a strong ability to induce osteogenic differentiation. The ARS staining showed that the SPEEK-GO surface exhibited significantly higher mineralisation ability than the other two groups. The results are consistent with the quantitative data.

## Discussion

While pure titanium and titanium alloy implant materials have long been used as materials for orthopaedic and dental implants [21], in recent years, patients have shown increasing resistance to the use of metal implants. As a result, PEEK has attracted attention for use in implants because of its excellent physical and chemical properties [22, 23]. Because the high incidence of implant failure is an important cause of peri-implant inflammation, we designed a PEEK implant surface with antibacterial properties that is expected to advance the development of antimicrobial implants.

In this study, due to 3D mesh sponge structure, GO nanosheets can be adsorbed to the surface, and the SEM results showed that GO uniformly covered the surface. Although a few pores were observed at low magnification, the GO nanosheet layer was overlaid on the surface of the SPEEK ultra-structure, and the sulfonated ultrafine fibres were wrapped by the GO nanosheets. Thus, it was observed that GO not only covered the material surface but was deeply embedded in the internal structure of the material. The GO nanosheet layer was strongly attached to the SPEEK ultra-structure, possibly due to the modification of the non-covalent interactions of PEEK after its sulfonation with concentrated sulfuric acid. After sulfonation, PEEK was unfolded and its π-π bonds were exposed, facilitating the formation of a strong π-π stacking bond between SPEEK and GO [24]. In addition, only the characteristic D and G peaks representing GO were observed in the Raman spectrum, and no signals of other chemical bonds were detected. This indicates that GO was successfully loaded onto the SPEEK surface, and the material surface was uniformly covered with GO. In addition, the wettability of SPEEK-GO was significantly increased, as demonstrated by the results of the contact angle measurements. This may be attributed to the large number of the oxygen-containing functional groups such as -OH and -COOH present on the GO surface.

In a previous study, Persson et al. [25] found that the occurrence of peri-implant inflammation may be related to the adhesion of *P. gingivalis* on implant or tooth surfaces. *P. gingivalis* is most closely related to the occurrence and development of peri-implant inflammation. The SPEEK-GO coating prepared in this experiment showed a significant decrease in the number of bacteria in the experimental group and a significant inhibition effect in both the plate-counting experiment and the SEM low-magnification image. Thus, it can be concluded that this coating can reduce bacterial adhesion and proliferation. However, the inhibition of *S. mutans* was slightly weaker than that of *P. gingivalis*. This may be because *P. gingivalis* is the Gram-negative bacteria with no cell wall and relatively weak resistance to external damage, while *S. mutans* is the Gram-positive bacteria that shows stronger resistance to external damage [26]. High-magnification SEM observations showed that most of the cells of *P. gingivalis* in the experimental group displayed wrinkling and deformation, while *S. mutans* showed cell membrane rupture and cell content overflow. These changes indicated a decrease in bacterial activity and were consistent with the results of the plate-counting assay, which showed that the number of bacteria in the experimental group was significantly lower. In a previous experimental study, these investigations were conducted using *S. aureus* and *E. coli* as the representative bacteria, rather than the common dental pathogens [24]. Therefore, the investigations performed in this work provide important information for the further development and use of GO as an antibacterial coating against the most common oral bacteria.

The development and severity of periodontitis and peri-implant inflammation are closely related to the Fim genotype carried by *P. gingivalis* strain [27]. In this study, the *Fim* gene in *P. gingivalis* was detected by RT-PCR, and it was shown that the SPEEK-GO surface significantly downregulated the relative expression of *Fim* in *P. gingivalis*. This may be attributed to the effect of the attack of the GO nanosheets on the bacterial cell membrane that prevented the production of *P. gingivalis* and the secretion of virulence factors. Extracellular glucans of *S. mutans* are mostly synthesised by the *Gtf* gene, a pathogenic gene that influences adhesion, biofilm formation, and the occurrence of dental caries [28]. Through a study on the relative expression level of the pathogenic genes, it was found that the downregulation of gene expression resulted in a decrease in the synthesis of water-insoluble glucan, reduced synthesis of ECM, and reduced bacterial adhesion. This result is consistent with the results of the plate-counting experiment in Figs. 7 and 9.

The adhesion and proliferation of cells are key to the formation of new bone on the surface of implants. Previous studies reported that the addition of GO on the biopolymer matrix could enhance cell attachment and proliferation via hydrophilic groups on the surface [29]. In this study, the CCK-8 method was used to detect the adhesion and proliferation of cells on the surface of different samples. Studies have previously demonstrated that the hydrophilicity of materials plays a crucial role in cell attachment and adherence [30]. High hydrophilicity provides a large surface area, which is conducive to interaction between protein molecules and cell-to-cell adhesion, thus enhancing the biological activity of biomaterials. The surfaces of SPEEK and SPEEK-GO were more hydrophilic than PEEK (Fig. 6), encouraging cell adhesion and proliferation (Fig. 12). The adhesion and proliferation of cells on the surface of the experimental group suggested high biocompatibility on the surface of the material, which is particularly important for the implantation material. The promotion of cell adhesion and proliferation of graphene materials may have occurred due to the accumulation of π-π stacking bonds between aromatic rings in the coating, which enabled proteins to attach quickly and provided a favourable environment for cell adhesion and proliferation [31].

It is well known that ALP activity is an early marker for osteoblastic differentiation on different specimens [32]. Our results showed that after 4 d of culture, the ALP activity of cells in the SPEEK-GO group was higher than that in other groups. As the culture time increased to 7 d, the ALP activity of SPEEK-GO group was significantly higher than that of the other groups, and the ALP activity of the pure PEEK group was the lowest, indicating that GO coating promoted early osteoblast differentiation, which is consistent with the conclusion of previous studies (Fig. 13). The mineralisation ability of MC3T3-E1 cells growing on different surfaces was quantitatively detected by ARS, and calcium nodules could be formed by reacting with calcium salt deposited extracellularly. The degree of osteogenic differentiation of different groups can be directly observed on the surface of the material. MC3T3-E1 cells were cultured for 14 d and qualitatively observed. Red calcified nodules were observed on the surface of SPEEK-GO and SPEEK, while almost no red calcified nodules were observed in the PEEK control group (Fig. 13).

## Conclusion

In this work, a porous SPEEK structure was successfully prepared, and the optimal sulfonation time was identified. The surface was subsequently successfully coated with GO using a simple deposition method. It was found that the hydrophilicity of the SPEEK-GO material surface greatly improved relative to that of the SPEEK samples. The SPEEK-GO coating was verified to exhibit strong antibacterial properties against the common dental pathogens *P. gingivalis* and *S. mutans*. At the same time, as an implant material, SPEEK-GO showed excellent cell compatibility and the ability to promote osteogenic differentiation. Therefore, this study provides a new method for the surface modification of GO onto porous PEEK to obtain enhanced antibacterial activity; the obtained SPEEK-GO material is a promising biomedical material, as it reduces the risk of implant-associated infections.
